# Modal lifespan and disparity at older ages by leading causes of death: a Canada-U.S. comparison

**DOI:** 10.1007/s12546-020-09247-9

**Published:** 2020-11-05

**Authors:** Viorela Diaconu, Nadine Ouellette, Robert Bourbeau

**Affiliations:** 1 Max Planck Institute for Demographic Research, Rostock, Germany; 2 Department of Demography, Université de Montréal, Montreal, QC Canada

**Keywords:** Mortality at older ages, Causes of death, Modal age at death, Lifespan inequalities, Canada, U.S.

## Abstract

The U.S. elderly experience shorter lifespans and greater variability in age at death than their Canadian peers. In order to gain insight on the underlying factors responsible for the Canada-U.S. old-age mortality disparities, we propose a cause-of-death analysis. Accordingly, the objective of this paper is to compare levels and trends in cause-specific modal age at death (*M*) and standard deviation above the mode (*SD*(*M* +)) between Canada and the U.S. since the 1970s. We focus on six broad leading causes of death, namely cerebrovascular diseases, heart diseases, and four types of cancers. Country-specific *M* and *SD*(*M* +) estimates for each leading cause of death are calculated from *P*-spline smooth age-at-death distributions obtained from detailed population and cause-specific mortality data. Our results reveal similar levels and trends in *M* and *SD*(*M* +) for most causes in the two countries, except for breast cancer (females) and lung cancer (males), where differences are the most noticeable. In both of these instances, modal lifespans are shorter in the U.S. than in Canada and U.S. old-age mortality inequalities are greater. These differences are explained in part by the higher stratification along socioeconomic lines in the U.S. than in Canada regarding the adoption of health risk behaviours and access to medical services.

## Introduction

International comparisons of life expectancy at birth or at a later age, and of lifespan variation—i.e., individual differences in the timing of death—revealed that the U.S. lags behind most industrialised countries (Barbieri and Ouellette 2012; Edwards and Tuljapurkar 2005; Engelman et al. 2010; Glei et al. 2010; White 2002; Wilmoth and Horiuchi 1999; Wilmoth et al. 2010; Wilson 2001). In comparison to Canada, the U.S. disadvantage in terms of survival time and inequality has been observed since the early 1960s and has gradually become more pronounced over time (Barbieri and Ouellette 2012; Edwards and Tuljapurkar 2005). The Canada-U.S. mortality differentials have also been observed at older ages: the U.S. elderly experience shorter lifespans and greater variability in age at death than their Canadian peers (Barbieri and Ouellette 2012; Glei et al. 2010; Ouellette and Bourbeau 2011).

**Fig. 1:**
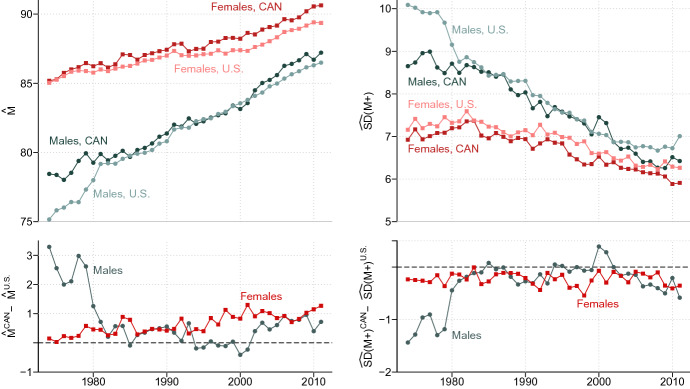
Estimated modal age at death, $\hat{M}$, and standard deviation above the mode, $\widehat{SD}$(*M* +), for all causes of death combined in Canada and the U.S., 1974–2011. Source: Authors’ calculations based on the Canadian Vital Statistics Death database, U.S. National Vital Statistics System data files, and Human Mortality Database

A key question has been whether the U.S. disadvantage at older ages is observable for all major causes of death, or are there specific causes for which the situation is reversed, i.e. the United States has an advantage over Canada? To answer this question, we compare levels and trends in cause-specific modal age at death, *M*, and standard deviation above the mode, *SD*(*M* +), between Canada and the U.S. over the period 1974–2011. We selected this mode-based pair of indicators because, as discussed later in the introduction, *M* is a lifespan measure that places special focus on survival improvements at older ages and it captures age shifts of old-age mortality more accurately than life expectancies at some selected old age (Horiuchi et al. 2013). Canada-U.S. differences in all-cause *M* and *SD*(*M* +) have already been reported by Ouellette and Bourbeau (2011). Figure 1 displays country-specific trends by sex from 1974 to 2011.

In the present article, we examine six leading causes of death among Canadian and U.S. elderly: cerebrovascular diseases, heart diseases, and four types of cancers, namely colorectal, lung, breast (females), and prostate (males) cancer. The present study is the first to conduct a Canada-U.S. comparison of patterns and trends in cause-specific modal age at death and standard deviation above the mode. We also provide the first available estimates in *M* and *SD*(*M* +) by leading causes of death in the U.S. (those for Canada were published in an earlier paper (Diaconu et al. 2016)). This comparative analysis aims to (1) determine whether the U.S. disadvantage with respect to old-age survival and lifespan variation holds for all six leading causes for the entire study period or only for specific causes and/or time periods and (2) examine whether the between-country difference with respect to cause-specific *M* and *SD*(*M* +) has narrowed or widened during the past four decades. Given that some causes are strongly related to health-related behaviours, such as lung cancer, while others are mostly amenable to medical care, such as prostate cancer, our results should provide insight on the factors responsible for the Canada-U.S. disparities in old-age mortality and uncover whether differences in health care systems stand out as the main culprit.

We focus on mortality changes at older ages because during the second half of the twentieth century, the extension of human life in high-income countries has been chiefly due to survival improvements at older ages, with the significant decline in mortality at ages 60 and above being its main contributor (Barbieri and Ouellette 2012; Mazui et al. 2014; Payeur 2011). In the last decades, the elderly have become a growing segment of the Canadian and U.S. population going from a level of about 10% in the early 1970s to about 15% in 2017 and are expected to reach 20–25% by 2030 (Statistics Canada 2012; US Census Bureau 2011; Vespa 2018). As increasingly more individuals survived to older ages the cause of death structure shifted from infectious diseases to chronic degenerative diseases. Heart disease, cancers, and stroke became the leading causes of death in developed countries, including in Canada and the U.S., since the 1960s.

Two demographic indicators, the adult modal age at death, *M*, and the standard deviation above the mode, *SD*(*M* +), have increasingly been used in the last decades for monitoring changes in the distribution of deaths at older ages in low mortality countries (Brown et al. 2008, 2012; Cheung and Robine 2007; Cheung et al. 2005, 2008, 2009; Diaconu et al. 2016; Kannisto 2007; Ouellette and Bourbeau 2011; Ouellette et al. 2012a, b; Robine and Cheung 2008; Thatcher et al. 2010), where the extension of the length of human life is primarily due to improvements in old-age survival (Meslé and Vallin 2006; Vallin and Meslé 2001; Wilmoth et al. 2010). Under a given mortality regime, *M* represents the most common (i.e., frequent) or ‘typical’ length of life among adults. Introduced in the nineteen century by Lexis (1877, 1878) as the most central and natural characteristic of human longevity, the adult modal age at death was rarely used by demographers until the early 2000s when it was reintroduced and popularised by Kannisto (2001) in human longevity studies.

*M*’s importance as a major indicator of old-age survival arises from a series of features, primarily as, unlike life expectancy at birth, *M* is solely influenced by old-age mortality (Canudas-Romo 2010; Horiuchi et al. 2013; Kannisto 2001). In fact, 2010 analytically showed that when mortality changes occur at younger ages but not at older ages, *M* remains unchanged. In the reversed situation, i.e. mortality reductions at older ages and no improvements at younger ages, *M* rises. For this reason, in many high-income countries, *M* remained constant or increased slightly over most of the first half of the twentieth century, when the increase in the length of human life was mainly due to survival im-provements among infants, children and young adults. However, throughout the second half of the twentieth century, mortality at older ages declined more rapidly than at younger ages, and *M* followed a steep upward trend (Canudas-Romo 2010; Cheung and Robine 2007; Cheung et al. 2009; Kannisto 2001; Office of National Statistics 2012). Another interesting feature of *M* was highlighted in a later study by Horiuchi and colleagues (2013), who provide empirical evidence and a mathematical proof that when mortality shifts to older ages, *M* increases at the exact pace as the old-age mortality shift while conditional life expectancy at some early old age, i.e. 50, 65, 75, increases more slowly. It should also be added that *M* has special mathematical properties, making for instance widely-used mortality models (e.g., Gompertz, logistic, Weibull) more clearly and straightforwardly understandable when *M* is used in replacement of the original mortality level parameter (Bergeron-Boucher et al. 2015; Horiuchi et al. 2013; Janssen and de Beer 2019; Missov et al. 2015).

With the reintroduction of the late modal age at death in contemporary demography, measures of variation relative to the modal age at death have also been proposed, the most frequently used being the standard deviation above the mode, *SD*(*M* +). The calculation of *SD*(*M* +) is based on deaths occurring beyond the modal age, such that a decline in *SD*(*M* +) over time indicates that deaths became increasingly concentrated into a shorter old-age interval beyond *M*, a phenomenon known as *old-age mortality compression*. In some high-income countries, the compression of mortality at older ages stalled in recent years, such as reflected by a constant *SD*(*M* +), while *M*’s upward trend continued. This phenomenon is known as the *shifting* mortality regime. Still, it should be noted that conceptually, according to Lexis’ (1878) concept of normal (i.e. gaussian) lifespans, the *SD*(*M* +) indicator is in- tended to measure the dispersion of senescent deaths around the modal age. Also, similarly to other measures of variation, *SD*(*M* +) can be used to inform on the degree of variability (inequality) in age at death across individuals.

## Data and methods

### Sources of data

Cause-specific mortality data for Canada and the U.S. are taken respectively from the Canadian Vital Statistics Death (CVSD) database of Statistics Canada and the U.S. National Vital Statistics System (NVSS) data files of the National Center for Health Statistics. In these two data sets, cause-specific death counts gathered from sub-national vital statistics registries are given by single years of age and sex since 1974 in Canada and since 1959 in the U.S. In the present paper, our analyses of levels and trends in cause-specific *M* and *SD*(*M* +) start in 1974 and end in 2011, covering three revisions of the ICD (i.e., the 8th, 9th and 10th revisions). Changes resulting from successive revisions of the ICD may create major discontinuities in cause-specific mortality trends over time, especially for highly detailed causes of death. The use of broad disease categories is less problematic. We thus focus on the following six broad leading underlying causes of death among males and females aged 10 years and above, in Canada and the U.S.: cerebrovascular diseases, heart diseases, and the four most diagnosed types of cancers, namely colorectal, lung, breast (females), and prostate (males) cancer. The concordance table used for bridging the three revisions of the ICD is provided in the “Appendix” (Table 1). We excluded individuals for which the age at death is unknown, as well as Canadians and non-U.S. residents deceased in the U.S. Their number accounts for less than 2% in each calendar year and country.

Canada and the U.S. are two rare countries where detailed high-quality data on deaths by underlying cause and single years of age over a long period of time are available. Because information is gathered at the individual level, the Canadian cause-specific mortality data are confidential, while the U.S. data still are freely available online. We were granted access to the Canadian data set through the Data Liberation Initiative, a program initiated by Statistics Canada to improve access to data resources for Canadian postsecondary institutions. Analyses made with the Canadian data are scrupulously verified before any analytic report is released or published in order to prevent the disclosure of any information deemed confidential.

These cause-specific mortality series from the CVSD and the NVSS are supplemented by estimates of population exposure by single years of age (10 and above), sex, and single calendar years (1974 to 2011) for Canada and the U.S., taken from the Human Mortality Database (HMD).

### The Poisson *P*-spline method for cause-specific mortality data

Within a cause-of-death framework, two functions characterise and describe the joint distribution of survival time *X* and type (cause) of death *K*: the cause-specific force of mortality and the cause-specific probability density function. Using sex-specific observed deaths counts by single years of age and cause of death as well as the population’s amount of exposure to the risk of dying at each age, these two mortality functions are estimated using a nonparametric smoothing technique known as *P*-splines (Eilers and Marx 1996). The Poisson *P*-spline method formerly introduced by Ouellette and Bourbeau (2011) for obtaining smooth age distributions of deaths (all causes combined) and for monitoring with great precision how these distributions have changed over time at older ages in low mortality countries was recently adapted to the context of cause-of-death analysis (Diaconu et al. 2016). Compared to mathematical mortality models for fitting age variations in adult mortality, namely the Gompertz, the logistic and the Weibull models, the *P*-spline method does not impose any assumptions related to the structure of the data. It has also been proven highly effective for fitting age-specific mortality rates and hence for obtaining smooth forces of mortality while remaining faithful to the specific characteristics of the observed data (Camarda 2008, 2012; Currie et al. 2004).

The smooth density function for cause *k*, describing the distribution of deaths across ages, is obtained from cause-specific smooth forces of mortality as follows:

$$\hat{f}_{k} \left( x \right) = \hat{\mu }_{k} \left( x \right)\hat{S}\left( x \right),$$

where $\hat{S}\left( x \right) = exp\left[ { - \mathop \smallint \limits_{0}^{x} {\hat{\mu }}\left( u \right)du} \right]$ and $\hat{\mu }\left( u \right)$ represents the all force of mortality estimated within a Poisson *P*-spline regression setting. For an illustration of cause-specific P-spline-smooth density curves see Fig. 4 for the U.S. in the “Appendix” (and Fig. A-2 in Diaconu et al. (2016) for Canada).

Under the assumption that causes of death are mutually exclusive and mutually exhaustive (Preston et al. 2001), the all-cause smooth force of mortality is derived from cause-specific smooth forces of mortality through summation.

The modal age at death for a given cause *k*, $\hat{M}_{k}$, indicating the age at which the highest proportion of deaths from this particular disease occurred, was obtained as follows:

$$\hat{M}_{k} = \mathop {\max }\limits_{x} \hat{{f_{k} }}\left( x \right).$$

As for its associated measure of dispersion, the standard deviation above the mode, $\widehat{SD}\left( {M_{k} + } \right)$, it is given by:

$$\widehat{SD}\left( {M_{k} + } \right) = \sqrt {{\raise0.7ex\hbox{${\mathop \smallint \nolimits_{{\hat{M}_{k} }}^{\omega } \left( {x - \hat{M}_{k} } \right)^{2} \hat{f}_{k} \left( x \right)dx}$} \!\mathord{\left/ {\vphantom {{\mathop \smallint \nolimits_{{\hat{M}_{k} }}^{\omega } \left( {x - \hat{M}_{k} } \right)^{2} \hat{f}_{k} \left( x \right)dx} {\mathop \smallint \nolimits_{{\hat{M}_{k} }}^{\omega } \hat{f}_{k} \left( x \right)}}}\right.\kern-\nulldelimiterspace} \!\lower0.7ex\hbox{${\mathop \smallint \nolimits_{{\hat{M}_{k} }}^{\omega } \hat{f}_{k} \left( x \right)}$}},}$$

where $\omega$ is the highest attained age at death.

## Results

### Canada-U.S. differentials in $\hat{\user2{M}}_{{\varvec{k}}}$

**Fig. 2:**
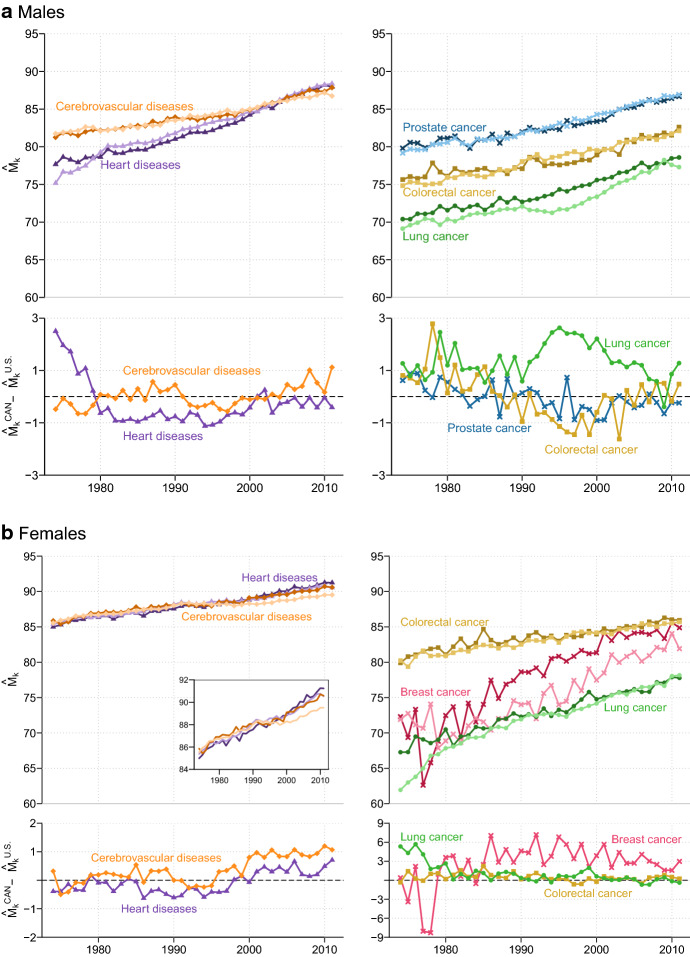
Estimated modal age at death, $\hat{M}_{k}$, for leading causes of death among the elderly in Canada (dark colors) and the U.S. (light colors) and corresponding country differences in $\hat{M}_{k}$ values, 1974–2011. *Note*: For calendar years 1977 and 1978 (in Canada), and 1974–1976, 1978 (in the U.S.), the smooth density function for breast cancer is bimodal. That is, we can distinguish two modal ages at death. In Fig. 2, only the “dominant” mode is illustrated, i.e., the age with the highest proportion of deaths. Please refer to Fig. 4 in the "Appendix" and to Figure A-2 in Diaconu et al. (2016) for an illustration of a smooth bimodal density function obtained with U.S. and Canadian data respectively. *Source*: Authors’ calculations based on the Canadian Vital Statistics Death database, U.S. National Vital Statistics System data files, and Human Mortality Database

Figure 2 depicts cause-specific trends and levels in the most frequent age at death, $\hat{M}_{k}$, among males (panel a) and females (panel b) in Canada and the U.S. over the period 1974–2011. In terms of trends, the figure firstly reveals that $\hat{M}_{k}$ increased substantially for all leading causes of death across sexes in both countries since the mid-1970s. Secondly, the pace of increase was nearly the same for most causes in the two countries, except for heart diseases (greater pace for males), breast cancer (greater pace for females), and lung cancer (blip in the early 1990s and greater pace in the 1970s for U.S. males and females only, respectively). In contrast, notable differences between the various causes of death studied are observable in terms of country-specific $\hat{M}_{k}$ levels. Males and females that died from lung cancer in Canada and in the U.S. exhibited the lowest modal age at death values while those who succumbed from cerebrovascular diseases, and from heart diseases in more recent years, had the highest values. Colorectal cancer’s modal ages at death ranked in between those for prostate and lung cancer among males, and slightly above those for breast cancer among females.

Looking at males’ trends in $\hat{M}_{k}$ for heart diseases and lung cancer closely, Fig. 2 (panel a) shows that in 1974, modal age at death was 75.2 (heart) and 69.1 (lung) years among U.S. males, ranking them 2.5 and 1.3 years behind their Canadian counterparts. For heart diseases, however, a crossover occurred in the early 1980s, allowing U.S. males to take the lead, but the gap then narrowed gradually and became barely noticeable by the end of the study period. In 2011, males died from heart disease in the U.S. and in Canada most frequently at around 88 years. For lung cancer, the Canada-U.S. gap in modal age at death remained quite stable in the beginning and in the end of the study period, with a clear dip for the U.S. somewhere in the middle. Indeed, from 1974 to 1990, $\hat{M}_{k}$ increased steadily and at a comparable pace in the two countries, but the level of the trend for Canadian males remained about 1 year higher than that of U.S. males. Then, $\hat{M}_{k}$ estimates for lung cancer among U.S. males declined slightly for about five consecutive years (due to a moderate increase in death rates for males in their early 70 s), while the upward trend of their Canadian peers continued uninterrupted. The gap in modal age at death between the two countries peaked at 2.6 years in 1995. In the following years, the gap narrowed and the pre-1990 upward trend resumed among U.S. males. In 2011 the most frequent age at death for lung cancer in American males was 1.3 years lower than their Canadian counterparts (77.3 vs. 78.6 years).

For causes other than heart diseases and lung cancer among males, differences in $\hat{M}_{k}$ levels between Canada and the U.S. are rather small. The modal age at death values for cerebrovascular diseases went from about 81 years in 1974 to 86 years in 2004, increasing at a similar pace in both countries for most of the study period. As of 2005, however, a small gap emerged between the two countries and became gradually more pronounced during the following years. In 2011, Canadian males dying from cerebrovascular diseases ‘typically’ survived about 1.2 years more than their American peers (87.9 vs. 86.7 years). For colorectal and prostate cancer, the U.S. modal age at death estimates show a subtle sinusoidal variation around the Canadian ones. Since 1974, the increase in $\hat{M}_{k}$ for both countries was about 7 years for the two types of cancer, reaching almost 82 and 87 years in 2011, respectively.

Among females the greatest disparities between Canada and the U.S. in $\hat{M}_{k}$ levels are recorded for lung cancer in the first years of the study period, and for breast cancer since the mid-1980s (Fig. 2, panel b). In 1974, the most frequent age at which lung cancer deaths occurred among U.S. females was about 5 years younger than for their Canadian counterparts (61.9 vs. 67.3 years). This prominent gap between the two countries was however observed for a short period of time only. Indeed, lung cancer’s $\hat{M}_{k}$ in the U.S. increased very rapidly from 1974 to the mid-1980s, reaching the Canadian levels by the early 1980s. From this point onwards, U.S. and Canadian females not only shared similar $\hat{M}_{k}$ levels but also followed a similar upward trend that culminated at nearly 78 years by 2011. In contrast, the modal age at death among Canadian and U.S. females dying from breast cancer were almost identical in 1974 (about 72 years) and no definite gap could be discerned between these countries over the next 10 $\hat{M}_{k}$ years. However, since the mid-1980s and until the end of the study period, a substantial survival advantage was observed for Canadian females. In 2011, the majority of deaths from this disease occurred at 85 years in Canada, or about 3 years later than in the U.S.

Canadian and U.S. female modal age at death estimates for the remaining causes displayed quite similar levels throughout the period 1974–2011. For the two main subcategories of cardiovascular diseases, $\hat{M}_{k}$ in 1974 was close to 85 years everywhere, but Canadian females started to record slightly higher levels for cerebrovascular diseases and, to a lesser extent, heart diseases in the mid-1990s and early-2000s, respectively. In 2011, Canadian females dying from heart or circulatory system condition survived until the year following their 90th anniversary, which is about 1 year later than their American peers. For colorectal cancer, females in Canada and the U.S. exhibited similar levels and patterns in $\hat{M}_{k}$ over the entire study period; they gained a little more than five years of ‘typical’ length of life since 1974, at an initial level of about 80 years.

In short, our close examination of sex- and cause-specific modal age at death estimates reveals that, with a few notable exceptions, Canada resembles greatly the U.S. in terms of pace of increase and level of $\hat{M}_{k}$ throughout the period under study. This resemblance applies to most causes of death, apart from lung cancer among males and breast cancer for females.

### Canada-U.S. differentials in $\widehat{{{\varvec{SD}}}}\left( {{\varvec{M}}_{{\varvec{k}}} + } \right)$

**Fig. 3:**
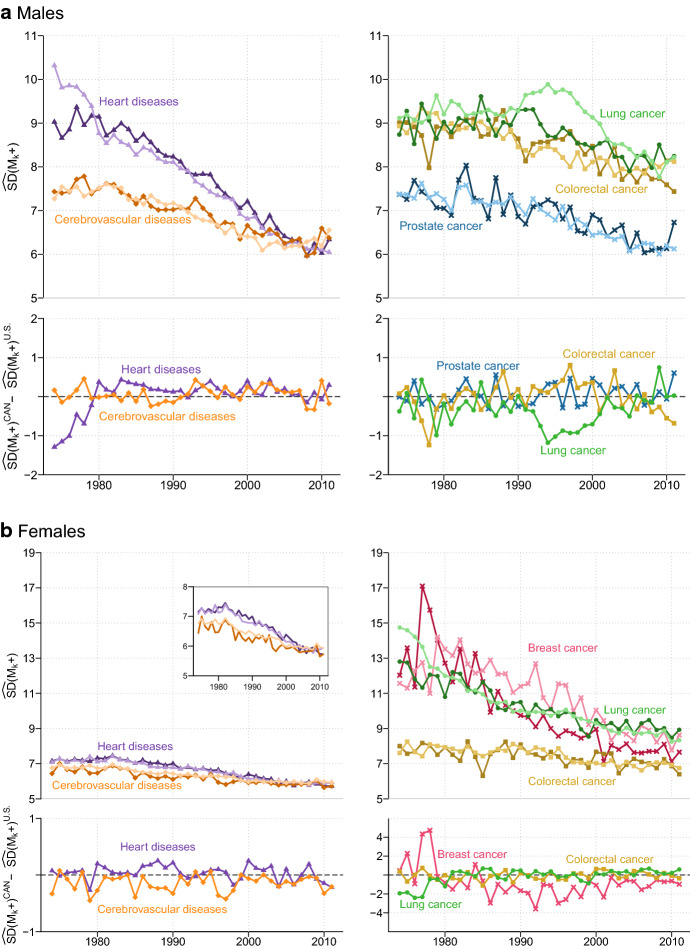
Estimated standard deviation of ages at death above the mode, $\widehat{SD}\left( {M_{k} + } \right)$, for leading causes of death among the elderly in Canada (dark colours) and the U.S. (light colours) and corresponding country differences in $\widehat{SD}\left( {M_{k} + } \right)$ values, 1974–2011. *Source*: Authors’ calculations based on the Canadian Vital Statistics Death database, U.S. National Vital Statistics System data files, and Human Mortality Database

**Fig. 4:**
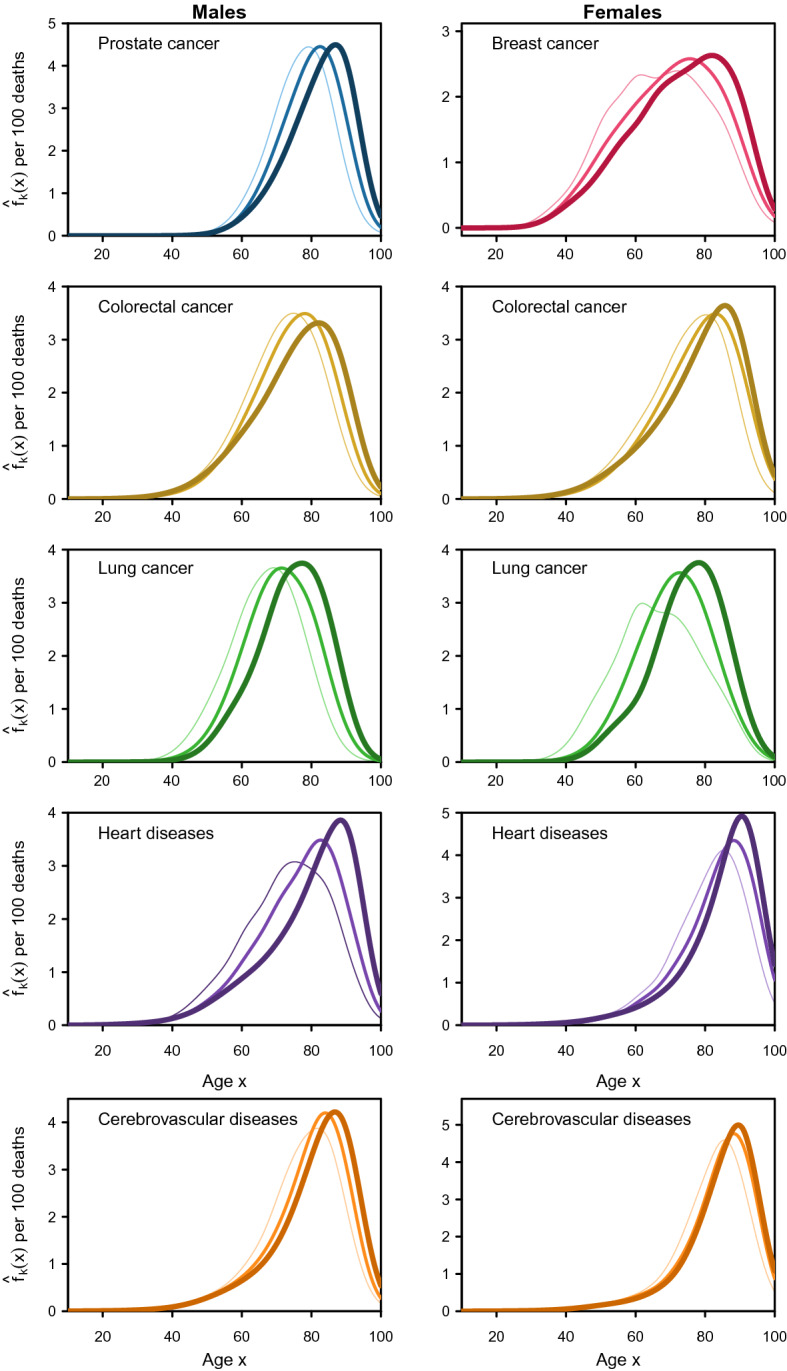
Smooth density functions describing the age-at-death distribution for leading causes of death among elderly U.S. males and females aged 10 and above, for calendar years 1974 (thin line), 1993 (medium line), and 2011 (thick line). *Source*: Authors’ calculations based on the U.S. National Vital Statistics System data files, and Human Mortality Database

Figure 3 shows cause-specific trends and levels in $\widehat{SD}\left( {M_{k} + } \right)$ among Canadian and U.S. males (panel a) and females (panel b) between 1974 and 2011. It reveals that $\widehat{SD}\left( {M_{k} + } \right)$ declined for all leading causes among Canadian and U.S. elderly since 1974. However, the causes of death differ greatly in terms of $\widehat{SD}\left( {M_{k} + } \right)$ levels. In general, male and female deaths from heart diseases are more dispersed across the old-age range than cerebrovascular diseases. The various types of cancers among males rank, according to their $\widehat{SD}\left( {M_{k} + } \right)$ values (from lowest to highest), as follows: prostate cancer, colorectal cancer, and lung cancer. Among females, colorectal cancer displayed the lowest $\widehat{SD}\left( {M_{k} + } \right)$ values in both countries with lung cancer, in Canada, and breast cancer, in the U.S., the highest.

Figure 3 (panel a) reveals similar levels in cause-specific $\widehat{SD}\left( {M_{k} + } \right)$ among males in both countries, except for heart diseases and lung cancer during specific time periods. For heart diseases, the highest Canada-U.S. difference in $\widehat{SD}\left( {M_{k} + } \right)$ was observed during the first 6 years of the study period. In 1974, variability of age at death above *M* was about 1.3 years higher in the U.S. than in Canada (10.3 vs 9 years respectively). The gap between the two countries rapidly narrowed following the accelerated decline in $\widehat{SD}\left( {M_{k} + } \right)$ in the U.S. By the early-1980s, variability of age at death above *M* in Canada was slightly higher than in the U.S. and remained this way throughout the rest of the study period. In 2011, variability in age at death among elderly Canadian males was a tad higher compared to their U.S. coun- terparts (6.3 and 6.0 years, respectively).

Substantial differences between Canadian and U.S. males were also noticed for lung cancer, especially during the 1990–2002 period. Until the late 1980s, variability of age at death among the elderly changed slightly in both countries (oscillated around 9 years). However, in the early 1990s a gap emerged between the two countries as $\widehat{SD}\left( {M_{k} + } \right)$ among U.S. males increased for about 4 years. During this period, $\widehat{SD}\left( {M_{k} + } \right)$ among Canadian males remained stable for about 2 years and declined afterwards. Hence, the greatest gap between the two countries occurred in 1994 when $\widehat{SD}\left( {M_{k} + } \right)$ reached 9.9 years in the U.S. and 8.7 years in Canada. Since the mid-1990s, $\widehat{SD}\left( {M_{k} + } \right)$ for lung cancer declined consistently in both countries attaining a similar level of about 8.2 years in 2011. Despite the identical level in $\widehat{SD}\left( {M_{k} + } \right)$ observed in both countries in more recent years, Canadian male deaths from lung cancer were in general concentrated into a shorter old-age interval compared to those of their U.S. counterparts for most of the study period.

For the remaining causes of death, the two countries exhibit small differences in terms of $\widehat{SD}\left( {M_{k} + } \right)$ levels. In addition, these countries experienced similar reductions in variability of age at death above *M*, for cerebrovascular diseases, prostate cancer, and to some extent colorectal cancer. In fact, $\widehat{SD}\left( {M_{k} + } \right)$ for cerebrovascular diseases and prostate cancer declined for about 1.4 years in both countries over the 1974–2011 period; going from a level of about 7.4 to 6 years. $\widehat{SD}\left( {M_{k} + } \right)$ for colorectal cancer, stagnated at a similar level of 9.0 years in both countries until 1989 and followed a downward trend in the years after. Since the early 1990s, U.S. elderly males registered lower lifespan inequalities than their Canadian peers, however a cross-over occurred in the early 2000s. Variability of age at death above *M* in 2011 was about 7 months higher in the U.S. than Canada (8.1 vs 7.4 years, respectively).

Canada-U.S. comparison of cause-specific $\widehat{SD}\left( {M_{k} + } \right)$ for females revealed small differences between the two countries for most leading causes of death, except for lung cancer, until late 1970s, and for breast cancer, since the 1980s (Fig. 3). In 1974, variability of deaths at ages above *M* for lung cancer in the U.S. was 14.7 years, that is about 2 years higher than in Canada. However, the $\widehat{SD}\left( {M_{k} + } \right)$ gap between the two countries narrowed rapidly and attained a level of about 4 months in 1981 as U.S. old-age variability levels converged towards the Canadian ones. For the remaining years of the study period, $\widehat{SD}\left( {M_{k} + } \right)$ exhibited similar trends and levels in both countries. In 2011, variability of age at death above M for lung cancer was slightly above 8.0 years in both countries. $\widehat{SD}\left( {M_{k} + } \right)$ for breast cancer fluctuated in Canada and in the U.S. for about 10 years before starting to steadily decline in the 1980s. Indeed, $\widehat{SD}\left( {M_{k} + } \right)$ among Canadian and U.S. females went respectively from about 13.3 and 12.1 years, in 1984, to 7.7 and 9.1 years, in 2011. Throughout most of this period, Canadian females exhibited lower levels of variability of age at death than their U.S. peers.

For the remaining causes, the gap in $\widehat{SD}\left( {M_{k} + } \right)$ levels between the two countries reached half a year at best throughout the 1974–2011 period. Unlike colorectal cancer for which almost identical $\widehat{SD}\left( {M_{k} + } \right)$ levels are observed in both countries, variability of age at death above *M* in Canada were slightly lower for cerebrovascular diseases and slightly higher for heart diseases compared to those in the U.S. In 1974, $\widehat{SD}\left( {M_{k} + } \right)$ for these three specific causes of death varied between 7.9 (colorectal cancer) and 6.4 years (cerebrovascular diseases) in Canada and between 6.8 (heart diseases) and 7.5 years (cerebrovascular diseases) in the U.S. The corresponding 2011 figures for these causes are respectively 6.4 and 5.7 years in Canada and 6.7 and 5.9 years in the U.S.

In sum, our analysis shows minor differences between Canada and the U.S. in terms of trends and levels in cause-specific $\widehat{SD}\left( {M_{k} + } \right)$. Two causes of death, however, exhibited a clear distinct pattern in Canada compared to the U.S. in terms of $\widehat{SD}\left( {M_{k} + } \right)$ trends: heart diseases (males only) and lung cancer (males and females). The differences were observed during specific time periods only, that is from 1974 to the early-1980s for heart diseases (males) and lung cancer (females) and from 1990 to 2002 for lung cancer (males).

## Discussion

This study has compared levels and trends in sex and cause-specific modal lifespan (*M*_*k*_) and disparity (*SD(M*_*k*_ +)) between Canada and the U.S. during the period 1974–2011. For many years since the mid-1970s, the U.S. had a mortality disadvantage relative to its northern neighbour with respect to these two lifespan indicators. Our paper investigated whether this U.S. old-age survival and inequality disadvantage was universal across the various leading causes of death examined, namely cerebrovascular diseases, heart diseases, colorectal cancer, lung cancer, female breast cancer, and male prostate cancer. The available evidence shows the contrary: the disadvantage was in fact due to specific causes at specific times only. We discuss this finding below, after offering our view on the otherwise similar trends and levels recorded in both countries.

The overall trends in modal lifespan are positive for all leading causes among males and females in Canada and the U.S. throughout the study period. The extension in the ‘typical’ length of human life was primarily due to improvements in old-age survival initiated by the “cardiovascular revolution”, which began around 1970 in most industrialised countries (Ouellette et al. 2014; Vallin and Meslé 2004). Following major breakthroughs in curative and preventive medicine, as well as changes in behavioural patterns and lifestyles brought upon by this revolution, cardiovascular mortality at adult and old ages started to decline steadily. The ongoing medical progress in the years that followed the onset of the cardiovascular revolution paved the way to a new wave of mortality reductions, but this time with respect to cancer. Indeed, the decline in cancer mortality observed in Canada and in the U.S. since the early 1990s (Cole and Rodu 1996; McLaughlin et al. 1997; Ouellette et al. 2014) has been primarily associated with advanced screening procedures, earlier detection of cancerous polyps, and effective therapeutic interventions (Cutler 2008; Edwards et al. 2005; Hankey et al. 1999; Karim-Kos et al. 2008; Mandel et al. 2000; Mariotto et al. 2002; Nam and Klotz 2009; NIH 1991; Schatzkin et al. 1994). Similar trends have also been observed for smoking-related cancers, in particular lung cancer, but the decline in mortality from this malignancy was mainly attributed to changes in patterns of smoking cessation (Devesa et al. 1989; Jemal et al. 2001) as well as reduced smoking uptake. The successful fight against cardiovascular diseases and the types of cancers studied here relied not only upon new biomedical knowledge and identification of risk factors, but also on individuals’ understanding and use of these preventive and curative developments. The ability of individuals to optimize their potential for a longer life has been notably associated with gains in educational attainment (Hayward et al. 2006; Hidajat et al. 2007). The changing educational composition of the Canadian and American populations since the 1970s has therefore certainly played a key role in lowering death rates for these diseases. As the educational attainment levels increased, individuals became more able to understand the benefits of new medical technologies and of healthier lifestyles.

While progress against cardiovascular and cancer mortality was being made, deaths that would have occurred at younger old ages were delayed to older old ages. This resulted in an increasing concentration of deaths in the age range beyond *M*, such as reflected by downward trends in cause-specific $\widehat{SD}\left( {M_{k} + } \right)$ since 1974 in the two countries. The compression of deaths into a shorter age span suggests narrower inequalities in the number of years lived by individuals dying within old age from these particular causes. In other words, individuals became increasingly similar in their capability of making informed decisions about their health and achieving longer lives. In addition to the benefits of rising educational attainment and declining educational differences between individuals, this capacity also appears to have been reinforced by health promotion campaigns, targeted to increase individuals’ knowledge regarding disease prevention. Indeed, in past decades, governments in North America notably have used various media campaigns to raise awareness within the population about the harmfulness of tobacco use, about heart disease avoidance, and about the importance of cancer screening (Wakefield et al. 2010). Another factor which likely played an important role in reducing lifespan variation at older ages is the greater capability of the health care system to meet individuals’ needs, both in terms of medical care via technological advancements in treating particular diseases and in increased means of providing health services to a larger segment of the population (Cohen et al. 2009; Easterlin 1996; Weatherall et al. 2006).

Levels in ${\hat{M}}_{k}$ and $\widehat{SD}\left( {M_{k} + } \right)$ between Canada and the U.S. were also highly similar for most causes of death studied (i.e. heart diseases, cerebrovascular diseases, colorectal cancer, male prostate cancer, and female lung cancer). This somewhat surprising finding may be a result of the differential comparative advantages of the Canadian and U.S. healthcare systems. While Canadians have a broader access to preventive care at all ages, there seems to be more aggressive medical treatment in the U.S., particularly at older ages when more Americans have access to insurance coverage. A study comparing U.S. all-cause agespecific death rates with those of 18 OECD countries, including Canada, indeed revealed that the U.S. ranked poorly at ages below 70 years but that its ranking improved dramatically after age 70 (Ho and Preston 2010). This reversal in ranking has been associated with the U.S.’s more aggressive treatment and use of drugs for curing diseases among the elderly. In particular for stroke and acute myocardial infarction, the U.S. has higher survival rates among individuals aged 65+ than in Canada, and the U.S. survival advantage grows with increasing age (Moise 2003; OECD 2003). Canadian patients diagnosed with acute myocardial infarction had a significant survival advantage over their American peers before undergoing revascularization. However, the survival gap between the two countries faded once a revascularization was performed (Kaul et al. 2004). The authors concluded that the U.S. survival advantage was due to the highly intrusive medical treatment provided to acute myocardial infarction patients. The U.S. is also more likely to resort to carotid endarterectomy, a surgical procedure which removes plaque from the carotid artery, for preventing death in individuals with high risk of stroke or recurrent stroke, than Canada (OECD 2003). Pharmacological treatments for reducing risk factors, such as hypertension, are also more aggressively used in the U.S. than in Canada. In fact, a higher percentage of older individuals (aged 50 and over) are taking antihypertensive drugs in the U.S. than in Canada (Crimmins et al. 2010; Wolf-Maier et al. 2014). Differences between the two countries have also been observed in the aggressiveness of the treatment regime provided to individuals diagnosed with prostate cancer. American urologists tended to use more intrusive screening methods and were also more likely to perform radical prostatectomy on older patients than their Canadian counterparts (Fleshner et al. 2000). In short, without a more intrusive medical approach in treating chronic diseases in the U.S. among the elderly, the U.S. would have ranked poorly with respect to old-age survival compared to Canada, as well as to other high income countries.

We found only two notable differences in modal age at death, ${\hat{M}}_{k}$, and standard deviation above the mode, $\widehat{SD}\left( {M_{k} + } \right)$, between Canada and the U.S, namely for lung cancer among males (throughout the period 1974–2011) and breast cancer among females (since the mid-1980s). In both cases, the U.S. showed a disadvantage compared with Canada by recording younger modal ages at death and greater variability around that mode.

With respect to lung cancer, the literature has identified prevalence and intensity of smoking as the primary sources of explanation for mortality differences across nations (Mackay et al. 2006; Pampel 2010). In the U.S., the percentage of adults who smoke and the cigarette consumption per smoker has been higher than in Canada since the 1980s (Ritchie and Roser 2020). The long-lasting U.S. lung mortality disadvantage with Canada among males in terms of modal age at death may also be explained in part by differences in smoking uptake. Studies show that individuals who never smoked have a much lower risk of dying from lung cancer than those who have (Anthonisen 2005; Doll et al. 2004; Ockene et al. 1990). In the U.S., residents aged 18 and over in 2002–2003 were interviewed and found to be more likely to be current smokers that their Canadian peers, who in contrast were more likely to be never smokers (Jones et al. 2010). The higher inequality in the age at death for lung cancer faced by U.S. elderly males compared to their Canadian counterparts may also stem from socioeconomic disparities in prevalence and intensity of tobacco use between the two countries. Indeed, in spite of similar levels of economic development and knowledge regarding the adverse health effects of cigarette consumption, developed nations differ with respect to smoking prevalence and intensity (Pampel 2010). We suspect that these differences are even more pronounced in countries displaying a wider economic divide between individuals from different social strata, given the strong association between smoking and socioeconomic status (Mackenbach et al. 2015; Tjepkema et al. 2012, 2013). Therefore, the larger income inequalities observed in the U.S. (Ross et al. 2000, 2005; Smeeding 2004) may be responsible for the higher levels of variability among U.S. males than Canadian ones.

The substantial gap in $\hat{M}$ and $\widehat{SD}\left( {M + } \right)$ levels for breast cancer puts U.S. females at a disadvantage. This result was somewhat unexpected because earlier studies have reported greater breast cancer survival rates in the U.S. than in Canada, thanks to more aggressive screening and treatment (Hughes 2003; Ugnat et al. 2005). The most compelling explanation for the U.S. old-age survival and lifespan inequality disadvantage for breast cancer may thus lie at the intersection of behavioural differences and social distribution mechanisms, such as accessibility of the health care system. Postmenopausal breast cancer has been associated with behavioural risk factors, including obesity and sedentary lifestyle (Levi et al. 2005; Menvielle et al. 2006; Yung and Ligibel 2016). While adult obesity prevalence increased in Canada and in the U.S. since the late 1970s, the gap between the two countries gradually widened with the highest proportion of overweight adult females being recorded in the U.S. (Alley et al. 2010). Also, because breast cancer is highly amenable to medical intervention through screening and therapy, it is possible that Canada and the U.S. differ with respect to access to these medical services. Studies comparing large metropolitan areas in Canada and the U.S. showed that Canadian women diagnosed with breast cancer and living in low-income areas have higher 5-year breast cancer survival rates than their U.S. counterparts. However, no significant Canada-U.S. differences were observed in the survival rates of women living in middle- and high- income areas (Gorey 2009; Gorey et al. 1997, 2009a). The Canadian advantage was observed even when women in the U.S. became eligible for Medicare (Gorey 2009). Therefore, differences in $\hat{M}_{k}$ for breast cancer may in part reflect the higher survival of Canadian women residing in less affluent areas compared to their American peers. This Canadian survival advantage has been associated to the more inclusive health care insurance coverage in Canada compared to the U.S., particularly among poorer women. Moreover, these studies have also revealed that unlike in Canada, there is a strong socioeconomic gradient in breast cancer survival in the U.S. (Gorey et al. 2000, 2009b). In the U.S. indeed, women diagnosed with breast cancer and living in high-income areas exhibited a higher survival advantage than their counterparts from middle-income areas, which in turn had higher survival chances compared to those residing in low-income areas. The country-difference in the magnitude of the socioeconomic gradient in breast cancer survival may in part be responsible for the Canada-U.S. gap in $\widehat{SD}\left( {M + } \right)$ levels.

In closing, while our study is the first to put forward the use of modal lifespan and disparity indicators in a cause-of-death analysis, we have yet to show how the cause-specific modal ages ($M_{k}$) contribute to changes in all-cause modal age (*M*), and similarly for the standard deviations above the mode (*SD*(*M* +) and $SD(M_{k} + )$). There are currently no methods which allow decomposing *M* or *SD*(*M* +) by cause of death.

## Appendix

See Table 1 and Fig. 4.

**Table 1:** Concordance table for bridging revisions 8, 9, and 10 of the ICD for leading causes of death in Canada and the U.S

Cause of death	ICD-8	ICD-9	ICD-10
Circulatory diseases	390–458	390–459	I00–I99
Cerebrovascular diseases	430–434, 436–438	430–434, 436–438	I00–I69
Heart diseases	390–398, 402, 404, 410–429	390–398, 402, 404, 410–429	I00–I09, I11, I13, I20–I51
Malignant neoplasms	140–208	140–208	C00–C97
Breast cancer	174	174	C50
Colorectal cancer	153–154	153–154	C18–C21
Prostate cancer	185	185	C61
Lung cancer	162	162	C33–C34

Items of ICD-8 for years 1969–1978, ICD-9 for 1979–1999, and ICD-10 since 2000 for Canada. Items of ICD-8 for years 1968–1979, ICD-9 for 1979–1998, and ICD-10 since 1999 for the U.S.

*Source*: Diaconu et al. (2016)
